# DNA-based vaccines: Advances, applications, and future prospects

**DOI:** 10.1016/j.gendis.2025.102025

**Published:** 2026-01-10

**Authors:** Zhichang Peter Zhou, Ziyan Rachel Chen, Ranmal Avinash Bandara, Rongqi Duan, Huibi Cao, Jun Liu, Jim Hu

**Affiliations:** a Translational Medicine Program, Hospital for Sick Children Research Institute, Toronto, Ontario M5G 0A4, Canada; b Department of Laboratory Medicine and Pathobiology, University of Toronto, Toronto, Ontario M5S 1A8, Canada; c Department of Molecular Genetics, Faculty of Medicine, University of Toronto, Toronto, Ontario M5S 1A8, Canada

**Keywords:** DNA vaccines, Immunotherapy, Infectious diseases, Pandemic, Plasmids, Viral vectors

## Abstract

DNA-based vaccines represent a promising advancement in immunization strategies, offering a novel approach for preventing infectious diseases and treating various conditions, including cancers and autoimmune disorders. These vaccines utilize genetically engineered plasmid or viral vector DNA encoding antigens of interest, which, upon administration, are taken up by host cells to produce the antigen *in situ*. This endogenous antigen expression elicits both humoral and cellular immune responses, mimicking natural infection pathways. Compared to conventional vaccines, DNA vaccines offer several advantages: they are relatively easy to design and manufacture, relatively stable, and capable of inducing long-lasting immunity without the need for live pathogens. Additionally, their platform is highly adaptable, enabling rapid development against emerging pathogens. Despite their success in animal models and promising results in clinical trials for infectious diseases caused by viruses, such as Zika, HPV, and SARS-CoV-2, DNA vaccines have faced challenges in eliciting robust immunogenicity in humans. Recent innovations—including improved delivery technologies, adjuvant formulations, and optimized plasmid and viral vectors—are addressing these limitations. The approval of DNA vaccines for veterinary use and recent human applications, such as Adenovirus (Ad) DNA-based Ebola vaccines and plasmid DNA-based COVID-19 vaccine, ZyCoV-D, mark significant milestones. Continued research and technological refinement are expected to expand their utility in global health. Overall, DNA-based vaccines hold great potential as a next-generation platform for safe, effective, and rapid-response immunization against a broad spectrum of diseases.

## Introduction

The field of vaccinology has undergone a remarkable transformation over the past few decades, driven by the emergence of innovative technologies that have expanded the scope and efficiency of vaccine development. Among these, DNA-based vaccines represent a groundbreaking approach that leverages genetic engineering to elicit protective immunity. First conceptualized in the early 1990s,^1^^,^^2^ DNA vaccines have progressed from theoretical constructs to viable candidates in both human and veterinary medicine.^3^^,^^4^ In most published literature, DNA-based vaccines are referred to as plasmid DNA-based vaccines. However, in this review, we also include virus-based DNA vaccines in this category. Unlike traditional vaccines, which rely on live-attenuated or inactivated pathogens or protein subunits, DNA vaccines employ DNA encoding target antigens. Upon delivery into host cells, the DNA is transcribed and translated, enabling the in vivo production of antigenic proteins that stimulate robust humoral and cellular immune responses. The literature search is performed systematically, such as for comparing different vaccine platforms, with work published in the last two decades. However, due to the large amount of work done in the field, especially since the recent pandemic, it is not feasible to cite all the related work in a single review article.

The COVID-19 pandemic catalyzed unprecedented investment and research in nucleic acid-based vaccines, resulting in the Emergency Use Authorization (EUA) of several mRNA vaccines^5^^,^^6^ and Ad vector-based DNA vaccines.^7^^,^^8^ Although DNA vaccines did not dominate early pandemic responses, their advantages—such as stability at ambient temperatures, low production costs, and ease of rapid design and scale-up—have positioned them as essential tools for future outbreaks. Notably, the approval of ZyCoV-D, a DNA vaccine for COVID-19 developed in India, marked a historic milestone as the first plasmid DNA vaccine authorized for human use.^9^^,^^10^ In addition, both the conventional Ad vector-based COVID-19 and mRNA-based vaccines contributed to mitigating the COVID-19 pandemic. Here, we directly compared COVID-19 vaccine platforms and highlighted the relative strengths and limitations of each platform (Table 1). The COVID-19 vaccines developed based on a more advanced Ad (helper-dependent Ad or high capacity) vector showed robust efficacies in animal models against live virus changes.^11^^,^^12^ These vaccines are expected to show better efficacy and safety profile in human studies compared to vaccines based on conventional Ad vectors; this will be discussed later in the section on delivery methods.

**Table 1:** Comparisons of COVID-19 vaccine platforms.

Vaccine	Platform	Efficacy	Immunological Outcomes	Storage	References
ZyCoV-D	DNA	66.6% (2 mg/dose,3-dose, 28 days apart)	Induces both humoral and cellular immunity. NAb GMT: 133 (day 84) IFN-γ: 48 SFC/million PBMCs	Primary storage (long-term use): 2 °C–8 °C. Short-term (3 months): 25 °C.	Khobragade et al,^9^ 2022
Ad26.COV2.S	Viral vector	75.2% (5 × 10^10^ vp/dose, 2-dose, 84 days apart).	Induces both humoral and cellular immunity, especially CD8^+^ T cells. Antibodies are relatively stable over 6 months. NAb GMT: 235 (28 days)	Supply: −25 °C to −15 °C. Primary storage (6 months): 2 °C–8 °C.	Zhang et al,^13^ 2022 Hardt et al,^14^ 2022 Sablerolles et al,^15^ 2022
AZD1222	Viral vector	66.7% (5 × 10^10^ vp/dose, 2-dose, 42–84 days apart)	Induces both humoral and cellular immunity NAb GMT:128–256 (day 84)	Supply and primary storage (6 months): 2 °C–8 °C.	Swanson et al,^16^ 2021
BNT162b2	mRNA	95% (2 doses, 21 days apart, 30 μg per dose.)	Induces both humoral and cellular immunity, especially NAb titers and CD4^+^ T cells. NAb GMT: 512 (30 days) and 64 (85 days). Antibodies wane over 6 months.	Supply and primary storage: −90 °C to −60 °C. Thawed storage: (10 weeks): 2 °C–8 °C.	Zhang et al,^13^ 2022 Polack et al,^6^ 2020 Levin et al,^17^ 2021
mRNA-1273	mRNA	94.1% (2 doses, 28 days apart, 100 μg)	Induces both humoral and cellular immunity, especially NAb titers and CD4^+^ T cells. NAb GMT: 69 (29 days) and 775 (119 days). Antibodies wane over 6 months.	Supply and primary storage: −50 °C to −15 °C. Thawed storage: (30 days): 2 °C–8 °C.	Zhang et al,^13^ 2022 Baden et al,^5^ 2021 Doria-Rose et al,^18^ 2021
NVX-CoV2373	Protein subunit	89.7% (3 doses, 21 days apart, 5 ug)	Induces both humoral and cellular immunity, especially CD4^+^ T cells. NAb GMT: 103.3 (21 days)	Primary storage (9 months): 2 °C–8 °C.	Zhang et al,^13^ 2022 Heath et al,^19^ 2021 Keech et al,^20^ 2020

Abbreviations.

NAb: Neutralizing Antibody.

GMT: Geometric Mean Titer.

PBMCs: Peripheral Blood Mononuclear Cells.

SFC: Spot-Forming Cells.

vp: Viral Particle.

Despite these advancements, DNA vaccines still face significant challenges, including relatively low immunogenicity in humans**,** poor delivery efficiency of plasmid DNA-based vaccines, and difficulty in scale-up production of helper-dependent Ad vector-based vaccines**.** Continued innovation in delivery technologies and improved antigen design are actively addressing these barriers.

This review provides a comprehensive overview of DNA-based vaccines, discussing their mechanisms of action, delivery strategies, clinical applications in infectious diseases and cancer, regulatory landscape, and recent technological innovations. Many previous review articles have covered plasmid DNA-based vaccines^21^, ^22^, ^23^ and this review will put more weight on viral vector DNA-based vaccines. By synthesizing current knowledge and highlighting future directions, this article aims to underscore the transformative potential of DNA vaccines in global health.

## Mechanisms of action

**Figure 1:**
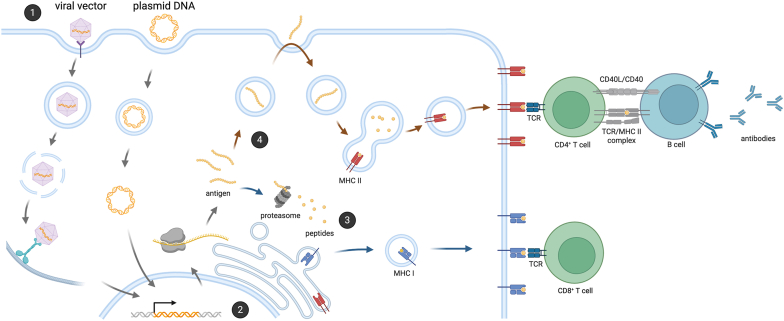
Mechanism of DNA-based vaccines. **1**. Viral or plasmid DNA enters the cell via endocytosis, followed by endosomal escape, which allows them to be transported into the nucleus. Some viruses specifically also recruit cytoplasmic dynein to transport towards the nucleus. **2.** The introduced DNA is transcribed in the nucleus and translated into antigenic proteins in the cytoplasm. **3.** Antigen processing via the MHC I pathway: the antigenic proteins are degraded by the proteasomes into peptides. Once these peptides are loaded on MHC I molecules, they can be presented on the cell surface and recognized by CD8^+^ T cells through T-cell receptors (TCR). Nucleated cells present antigen in this pathway. Upon activation, the CD8^+^ T cells differentiate into cytotoxic effector cells. **4.** Antigen processing via the MHC II pathway: antigens can be secreted via exosomes. The extracellular or endocytosed antigen is processed in the endosomes and loaded on MHC II molecules. The MHC II-peptide complexes are then presented on the cell surface (of professional antigen-presenting cells, like dendritic cells, macrophages, and B cells) to activate CD4^+^ T cells. Their interaction with B cells ultimately leads to antibody production.

DNA-based vaccines work by harnessing the host's cellular machinery to produce an antigenic protein that elicits an adaptive immune response. Unlike traditional vaccines that deliver inactivated pathogens or protein subunits, DNA vaccines introduce genetically engineered plasmid or viral DNA encoding the antigen of interest. As illustrated in Figure 1, the processes involve several key steps.

### DNA uptake and translocation

Following administration, typically via intramuscular or intradermal injection, DNA enters host cells, predominantly muscle cells and local antigen-presenting cells (APCs). While the exact mechanism is not fully understood, plasmid or viral vector internalization is thought to occur through endocytosis or transient membrane disruption. This process may not be very efficient because DNA needs to escape from endosomes and be transported into nuclei. However, in some viruses, for example, Ad, the viral capsid proteins can help them escape from endosomes and enter the nucleus through dynein-assisted transport.^24^^,^^25^

### Transcription and translation of antigen

Once inside the nucleus (but not integrated into the host genome), the plasmid or viral vector DNA is transcribed into messenger RNA (mRNA) by the host's RNA polymerase II. The resulting mRNA is then exported to the cytoplasm, where ribosomes translate it into the encoded antigenic protein. These antigens can be derived from viral, bacterial, or tumor-associated proteins.

### Antigen processing and presentation

The expressed protein antigens undergo intracellular processing via two main pathways. One is the endogenous pathway where intracellular proteins are degraded by proteasome, and peptides are presented on major histocompatibility complex (MHC) class I molecules. This pathway is crucial for activating cytotoxic CD8^+^ T lymphocytes (CTLs), which target and eliminate infected or abnormal cells. The other is the exogenous pathway (cross-presentation), where some of the expressed antigen is secreted or released from dying cells and taken up by professional APCs such as dendritic cells. These antigens are processed and presented on MHC class II molecules, leading to the activation of CD4^+^ T helper cells and subsequent stimulation of B cells for antibody production.

### Activation of adaptive immune responses

The antigen presentation on MHC molecules results in the activation of both arms of the adaptive immune system: Cellular immunity where CD8^+^ T cells are primed to recognize and kill target cells presenting the antigen, and humoral immunity where activated CD4^+^ T cells provide help to B cells, which differentiate into plasma cells that produce antigen-specific antibodies. However, T cell activation not only requires antigen presentation, but also an environment with the presence of certain proinflammatory cytokines, such as interferon (IFN)-γ, IFN-α/β, interleukin (IL)-1β, IL-2, IL-4, and IL-12. In viral vector delivery of vaccines, the viral proteins and DNA can promote the expression of these cytokines, so that there is no need to use adjuvants for the vaccination. For plasmid-based DNA vaccines, adjuvants are normally required for the vaccination. Table 2 lists all the adjuvants used for vaccine delivery. Memory T and B cells generated during the primary immune response induced by effective vaccines can persist and provide rapid and robust protection upon subsequent exposures to the pathogen.

**Table 2:** Adjuvants used for plasmid DNA-Based vaccines.

Adjuvant Type	Adjuvant Name	Function/Mechanism	References
Cytokine adjuvants	IL-2, IL-12, GM-CSF	Enhance T-cell responses and antigen presentation	Kim et al,^26^ 2000; Zhang et al,^27^ 2011
TLR Agonists	CpG ODN (TLR9 agonist)	Stimulate innate immune response via TLR9	Verthelyi et al,^28^ 2002
	Poly(I:C) (TLR3 agonist)	Induce type I interferons, enhance Th1 responses	Trumpfheller et al,^29^ 2008
Nano-formulations	Liposomes, lipid nanoparticles	Protect plasmid DNA and enhance cellular uptake	Perrie et al,^30^ 2002; Fotoran et al,^31^ 2017
	Cationic polymers (*e.g.*, PEI)	Facilitate cell entry, endosomal escape	Singh et al,^32^ 2000
Emulsions	Montanide ISA 51 and 720	Depot effect, slow antigen release	Aucouturier et al,^33^ 2002
Mineral salts	Aluminum hydroxide (limited use)	Traditional adjuvant; used less due to poor Th1 stimulation	Liao et al,^34^ 2023
Saponins	QS-21	Induce strong cellular and humoral immunity	Clark et al,^35^ 1991
Heat-labile enterotoxins	LT, CT subunits	Mucosal adjuvants for intranasal/oral DNA vaccines	Arrington et al,^36^ 2002
Bacterial derivatives	Flagellin (TLR5 agonist)	Activate innate immunity via TLR5	Applequist et al,^37^ 2005
STING agonists	cGAMP	Activate STING pathway, promote type I IFNs	Padron-Regalado et al,^38^ 2022; Ulrich-Lewis et al,^39^ 2002
Electroporation	CELLECTRA	Enhances DNA uptake and local inflammation	Tebas et al,^40^ 2021

### Innate immune activation

In addition to adaptive immunity, DNA vaccines can stimulate the innate immune system through the recognition of foreign DNA by pattern recognition receptors (PRRs) such as Toll-like receptor 9 (TLR9) in plasmacytoid dendritic cells. The activation of the innate immune system establishes a so-called trained immunity where innate immune cells (such as monocytes, macrophages, γδ T cells, and natural killer cells) exhibit a memory-like response after an initial stimulus, leading to an enhanced response upon re-exposure to the same or even unrelated pathogens.^41^, ^42^, ^43^

## Advantages of DNA vaccines

DNA vaccines offer several distinct advantages over conventional vaccine platforms, making them an attractive strategy for the prevention and treatment of infectious diseases, cancers, and other conditions. Their versatility, stability, and ability to elicit broad immune responses are particularly notable.

### Safety profile

DNA vaccines are non-infectious and do not use live pathogens, reducing the risk of reversion to virulence or unintended infection. Additionally, because the plasmid and viral vector DNA remain episomal and do not integrate into the host genome, the risk of insertional mutagenesis is extremely low. For optimized design, a cDNA encoding a dominant antigenic protein of a pathogen, such as the spike protein of SARS-CoV-2, can be selected as an antigen and tested in animal models to avoid antibody-dependent enhancement (ADE). ADE is a major concern for vaccine development because in vaccinated individuals with ADE, antibodies enhance rather than protect against viral infection.

### Ease and speed of design

**Figure 2:**
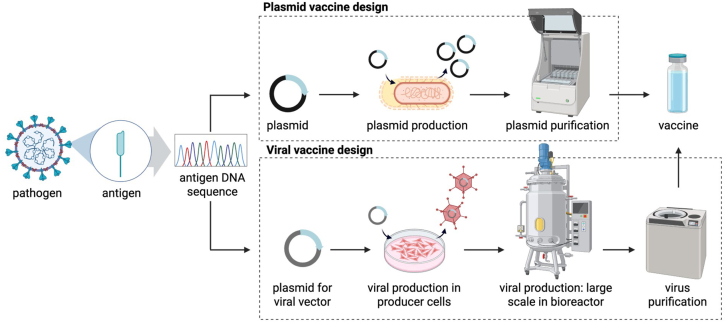
Schematic workflow of DNA vaccine design. Once the antigen DNA sequence has been determined, it is cloned into an expression plasmid. For plasmid-based vaccines, the plasmids are propagated through bacterial fermentation and subsequently purified. For viral-vector vaccines, the antigen-containing plasmids are transfected into producer cells, enabling the assembly of viral particles expressing the antigen. The resulting viral vectors are then amplified and purified to generate the final vaccine product.

The schematic workflow of DNA vaccine design is shown in Figure 2. DNA vaccine constructs can be rapidly generated once the genetic sequence of the target antigen is known. This speed of development is particularly advantageous in responding to emerging infectious diseases, such as during the COVID-19 pandemic. Plasmid or viral DNA can be easily engineered to encode multiple antigens or antigenic domains, enabling the development of multivalent vaccines that target several strains or pathogens simultaneously. This modularity allows precision tailoring of immune responses.

### Induction of both humoral and cellular immunity

DNA vaccines are capable of inducing robust B cell (antibody-mediated) and T cell (cell-mediated) responses. Importantly, they can elicit strong cytotoxic CD8^+^ T cell responses, which are critical for controlling intracellular pathogens and tumors. Meanwhile, DNA vaccines also induce B cell response, contributing to comprehensive immune protection.

### Scalability and low-cost manufacturing and storage

Production of plasmid DNA vaccines involves bacterial fermentation and plasmid purification, processes that are relatively low-cost, scalable, and do not require cell cultures or bioreactors for antigen production. Thus, it is highly cost-effective, especially at a large scale. For most viral DNA-based vaccines, the production in well-established packaging cell lines is highly cost-effective. DNA plasmids and viruses are inherently stable and resistant to temperature fluctuations, unlike many protein-based or mRNA vaccines. This facilitates easier distribution and stockpiling, especially in resource-limited settings.

### Suitability for therapeutic applications

In addition to prophylactic use, DNA vaccines have potential in therapeutic settings, particularly in cancer immunotherapy, where they can be designed to express tumor-associated antigens and stimulate anti-tumor T cell responses.^22^^,^^44^

## Delivery methods

**Figure 3:**
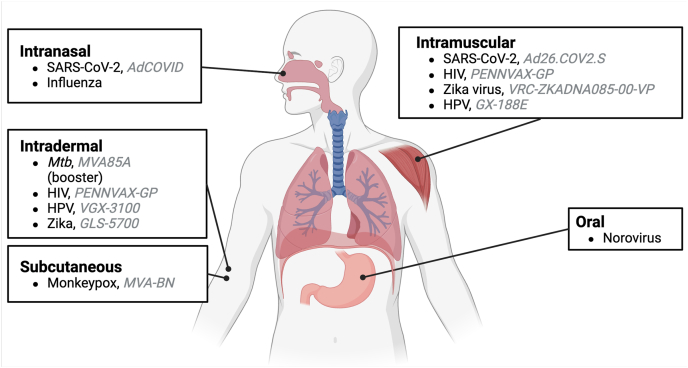
Routes of vaccine delivery. Vaccines can be administered via intranasal, intradermal, subcutaneous, intramuscular, and oral routes. Examples of target pathogens and their corresponding vaccine candidates (where applicable) are provided for each route.

Efficient delivery of DNA into host cells is a critical determinant of the immunogenicity and overall success of DNA-based vaccines. Both viral^45^, ^46^, ^47^ and non-viral^48^^,^^49^ vehicles have been used in the delivery of DNA-based vaccines. The most common administration route of the vaccination is intramuscular injection while other routes, such as intradermal injection^10^ or aerosolization via airway,^12^ can also be used (Fig. 3). Because plasmid DNA is susceptible to degradation and has limited cellular uptake, various physical and chemical delivery strategies have been developed to enhance transfection efficiency, antigen expression, and immune activation. However, virus DNA is protected by viral capsid proteins and has efficient cellular uptake, rendering more efficient delivery. Below are the major delivery approaches based on viral or non-viral vehicles employed.

### Viral vector delivery

Several types of DNA viruses have been used for vaccine delivery (Table 3). Viral vectors can induce proinflammatory cytokine expression to enhance vaccine-induced immunity; therefore, adjuvants are dispensable for vaccine delivery.^50^ Because Ad vectors have been extensively explored for their utility in vaccine delivery, they are used here as an example. The Ad vectors are genetically modified from natural adenoviruses, which are non-enveloped viruses with linear double-strand DNA genomes ranging from 26 to 45 kilobases (kb), depending on the serotypes. Based on the extent of viral genome deletion, Ad vectors are categorized into three generations. The first-generation vectors are modified by deletion of the early region 1 (E1) and/or E3. As a result, the vectors are replication-defective, and exogenous DNA up to a size of 8.2 and/or 5.1 kb can be inserted into the vectors.^51^ The second-generation vectors are created by further deleting E2 and/or E4 genes, expanding exogenous DNA capacity to 14 kb.^52^ The third-generation vectors, also known as helper-dependent Ad (HDAd) or gutless Ad vectors, are devoid of all viral genes except the inverted terminal repeats and packaging signals.^53^ This design minimizes viral gene expression and makes HDAd vectors less toxic than other generations. The HDAd vectors have a large capacity of 36 kb of exogenous DNA. Because Ad vectors can be produced at a large scale and induce strong cellular and antibody responses, they are among the most popular choices for vaccine development.^7^^,^^8^^,^^12^ One challenge in using Ad vectors (and possibly other viral vectors) for delivery is the limited boosting effect when the same vector type is used repetitively, likely due to immunological memory formed from the prime dose. One solution is to use heterologous vectors for the boosting dose, as exemplified by the Ebola vaccine, Zabdeno/Mvabea, developed by Johnson and Johnson,^54^^,^^55^ and the COVID-19 vaccine developed by Gamaleya Institute.^56^

**Table 3:** DNA viruses used for vaccine delivery.

Virus Type	Genome Type	Key Features	Use in Vaccines	Application Examples in Human	Reference(s)
Adenovirus (Ad)	dsDNA	Attenuated/non-replicating, large cloning capacity, high immunogenicity, well-studied	Clinical/experimental	Clinical: COVID-19 (JCOVDEN), Ebola (Ad26.ZEBOV) experimental: TB, Malaria, Zika	Sadoff et al,^47^ 2022; Pollard et al,^57^ 2021;
					Palma et al,^58^ 2007 Sankaradoss et al,^59^ 2022
Poxvirus (MVA, ALVAC)	dsDNA	Attenuated/non-replicating, large cloning capacity, strong immune response	MVA, clinical/experimental	Clinical: Smallpox (Jynneos), Ebola (MVA-BN-Filo) experimental: TB	Deputy et al,^60^ 2023; Puri et al,^61^ 2024;
					Tameris et al,^62^ 2013
			ALVAC, experimental	HIV	Costanzo et al,^63^ 2023
Herpesvirus (CMV)	dsDNA	Long-term expression, large genome	Experimental	TB, Malaria	Hansen et al,^64^ 2018; Hansen et al,^65^ 2019
Parvovirus (AAV)	ssDNA	Safe, low immunogenicity	Experimental	Gene therapy, some vaccine studies	Nelson et al,^66^ 2019; Demminger et al,^67^ 2020
Baculovirus	dsDNA	Non-replicating in mammals, safe	Clinical/Experimental	Clinical: protein production for subunit vaccines (FluBlok, Novavax)	Baxter et al,^68^ 2011; Heath et al,^19^ 2021
				Experimental: DNA delivery	Capin et al,^69^ 2024

To improve the viral vector delivery of vaccines, gutted viral vectors (viral coding sequences deleted) should be considered because the deletion of viral coding sequences will improve the expression of antigens and the safety profile of the vaccines. During the COVID-19 pandemic, our team developed vaccines using the helper-dependent viral vector and demonstrated potent protective effects against live viral challenge in animal models.^11^^,^^12^

### Non-viral vector delivery

Naked DNA is susceptible to degradation in cells and has poor nuclear uptake. Therefore, physical or chemical approaches are needed to enhance the delivery of plasmid DNA-based vaccines. Table 4 lists various strategies to enhance the delivery of plasmid DNA-based vaccines. Some of these strategies will be further discussed later.

**Table 4:** Non-viral approaches for DNA-based vaccine delivery.

Category	Method	Mechanism	Representative Reference
Physical	Electroporation	Electrical pulses increase cell membrane permeability	Sardesai et al, 2011^70^
Physical	Gene gun (Biolistic)	DNA-coated particles propelled into cells by high-pressure gas	Barry et al, 2000^71^
Physical	Microneedles	Minimally invasive delivery to skin immune cells	Kim et al, 2012^72^
Physical	Jet injection	High-velocity stream delivers DNA intradermally or intramuscularly	Jiang et al,^73^ 2019
Physical	Ultrasound (Sonoporation)	Ultrasound temporarily opens membrane pores	Miller et al,^74^ 2002
Chemical	Cationic lipids (Lipoplexes)	Lipid-DNA complexes fuse with membranes	Li and Petrovsky,^75^ 2016
Chemical	Cationic polymers	Polymers like PEI or chitosan form polyplexes with DNA	Pack et al,^76^ 2005
Chemical	Dendrimers	Branched polymers with high DNA binding capacity	Daftarian et al,^77^ 2011
Chemical	Peptide-based delivery	Cell-penetrating peptides facilitate DNA entry	Torchilin,^78^ 2008
Chemical	Lipid–Polymer hybrids	Combines liposome and polymer advantages for DNA delivery	Li et al,^79^ 2022
Emerging systems	Hydrogels	Slow-release matrices protect and deliver DNA	Tian et al,^80^ 2014
Emerging systems	DNA Nanostructures	Self-assembling programmable DNA carriers	Franck et al,^81^ 2021
Emerging systems	Exosomes	Natural vesicles used for biocompatible DNA transfer	El Andaloussi et al,^82^ 2013

## Applications of DNA-based vaccines

DNA-based vaccines have rapidly evolved from a novel experimental concept to a versatile platform with broad applications across infectious diseases, oncology, and beyond. Their adaptability, rapid design potential, and ability to elicit both humoral and cellular immunity make them suitable for both prophylactic and therapeutic purposes. While there is a comprehensive review has already summarized DNA vaccines evaluated in Phase I and II clinical trials,^83^ here we focus on the most clinically advanced candidates. Accordingly, in Table 5, we have summarized major DNA vaccines that have entered Phase III clinical trials.

**Table 5:** Phase III clinical trial outcomes of representative DNA and viral vector-based vaccines.

Vaccine	Developer	Diseases	Vector/Delivery	Efficacy Findings	Safety	References
Plasmid DNA						
ZyCoV-D	Cadila Healthcare	COVID-19	pVAX1 plasmid; intradermal (needle-free PharmaJet Tropis® ID).	66.6% (See Table 1 for details)	Injection site reactions: pain, redness, swelling, and itching. Systemic reactions: fatigue, headache, muscle pain, nausea, and fever.	Khobragade et al,^9^ 2022
INO-4800	Inovio Pharmaceuticals	COVID-19	pGX-series plasmid; intradermal and plus electroporation (CELLECTRA® EP).	Efficacy not reported (2 mg/dose, 2-dose, 28 days apart). NAb GM T: 6.6 (day 58 IFN-γ: 162 IFN-γ-secreting cells/million PBMCs	Injection site reactions: pain, redness, swelling, and itching. Systemic reactions: fatigue, headache, fever, myalgia, and nausea.	Jia et al,^84^ 2025
VGX-3100	Inovio Pharmaceuticals	HPV-16/18 related cervical HSIL	pGX-series plasmid; intramuscular; CELLECTRA® EP.	48.2% *vs* 30% (placebo) achieved histopathological regression (6mg/dose, 3-dose, 28 and 84 days apart).	Injection site reactions: pain, redness, swelling, and itching. Systemic reactions: Headache, fatigue, fever.	Daftarian et al,^77^ 2011
Viral vector						
AZD1222	University of Oxford/AstraZeneca	COVID-19	ChAdOx1 (chimpanzee Ad); intramuscular.	66.7% (See Table 1 for details)	Injection site reactions: pain, redness, swelling systemic reactions: fatigue, headache, and myalgia. Rare cases: Thrombosis with thrombocytopenia syndrome	Voysey et al,^85^ 2021 Bhuyan et al,^86^ 2021
Ad26.COV2.S	Janssen (Johnson & Johnson)	COVID-19	Ad26 (human Ad, type 26); intramuscular.	75.2% (See Table 1 for details)	Injection site reactions: pain, redness, swelling systemic reactions: fatigue, headache, and myalgia. Rare cases: Thrombosis with thrombocytopenia syndrome	Hardt et al,^14^ 2022 Sablerolles et al,^15^ 2022 Muir et al,^87^ 2021
Sputnik V	Gamaleya research Institute	COVID-19	Heterologous (Ad26 + Ad5); intramuscular.	91.6% (10^11^ vp/dose, 2-dose, 21 days apart). NAb GMT 44.5 (42 days)	Injection site reactions: pain, redness, swelling systemic reactions: fatigue, headache, and myalgia.	Logunov et al,^88^ 2021
Viral vector (replicating)						
Ervebo (rVSV-ZEBOV)	Merck	Ebola virus	rVSV; intramuscular	100% (7.2 × 10^7^ PFUs, 1 and 2-dose). NAb GMT (28 days): 277.1 (children) and 169.2 (adults)	Injection site reactions: pain, redness, swelling Systemic reactions: fatigue, headache, and myalgia. Rare cases: Anaphylaxis	Meakin et al,^89^ 2024

**Abbreviations**.

SAEs: Serious Adverse Events.

NAb: Neutralizing Antibody.

GMT: Geometric Mean Titer.

SFC: Spot-Forming Cells.

PBMCs: Peripheral Blood Mononuclear Cells.

vp: Viral Particle.

PFUs: Plaque-Forming Units.

HSIL: High-Grade Squamous Intraepithelial Lesion.

rVSV: Recombinant Vesicular Stomatitis Virus.

Below are key areas where DNA vaccines are currently applied or under active investigation.

### Infectious diseases

One of the most prominent and well-established uses of DNA vaccines is for preventing infectious diseases. By encoding antigenic proteins from pathogens, DNA vaccines can induce protective immune responses without the need for live or attenuated organisms.

**Viral** **infections**: DNA vaccines have been developed for a wide range of viruses, including influenza,^90^ human papillomavirus (HPV),^91^ Zika virus,^92^^,^^93^ dengue,^59^^,^^94^ HIV,^95^^,^^96^ and SARS-CoV-2.^7^^,^^8^ While most of these vaccines remain in early-phase or preclinical studies, some have made significant progress. One example is VGX-3100 for treating cervical dysplasia. It is a plasmid-based vaccine targeting the E6 and E7 proteins of HPV-16 and HPV-18, and it can be administered through muscular electroporation. The VGX-3100 has demonstrated efficacy against cervical intraepithelial neoplasia in a recent phase 2b trial.^91^ Although VGX-3100 failed to meet the primary endpoint in the Phase III REVEAL-2 trial (ref, *Inovio Pharmaceuticals. REVEAL-1 Phase III trial results for VGX-3100*), the limited efficacy was attributed to the biomarker-selected trial design, which narrowed the responsive population, and potentially to suboptimal T cell responses in certain individuals. By using data from the broader “all-participants” endpoint (which included all patients, not just those with the biomarker), VGX-3100 did show statistically significant improvement in lesion regression and viral clearance compared to the placebo.

Another example is the ZyCoV-D DNA vaccine against SARS-CoV-2. ZyCoV-D encodes the spike protein of SARS-CoV-2, along with an IgE signal peptide, and can be administered with a needle-free injection system. The Phase I/II study, an open-label, non-randomized trial in healthy adults, demonstrated that the vaccine was safe, well-tolerated, and capable of inducing robust immune responses.^10^ Notably, by day 84, in the 2 mg dose group, all individuals administered via a needle-free injection system developed higher specific serological responses than those vaccinated with needles. Specifically, the geometric mean titer of IgG was 126.29 versus 106.92, and the neutralizing antibody titer was 7.83 versus 2.83. In addition, cell-mediated response was detectable by day 84, with IFN-γ levels reaching 45.5 SFC per million peripheral blood mononuclear cells in the 2 mg dose group. Building on this, the Phase III randomized, double-blind, placebo-controlled trial in India evaluated the vaccine efficacy, safety, and immunogenicity in a larger population.^9^ The interim analysis showed the vaccine efficacy was 66.6% against COVID-19, with similar adverse event rates in the vaccine (4.49%) and placebo (4.47%) groups. Together, these studies indicate that ZyCoV-D is a safe and effective DNA vaccine capable of providing moderate protection against SARS-CoV-2 infection.

Another example is the well-known Ad-based COVID-19 vaccines. In the early phase of the COVID-19 pandemic, four Ad-based vaccines were approved for emergency use. These vaccines are based on human Ad26,^8^^,^^56^ human Ad5,^56^^,^^97^ or chimpanzee Ad^7^ serotype, and typically encode the full-length SARS-CoV-2 spike protein, with or without stabilizing mutations. Compared to mRNA-based vaccines, Ad-based vaccines generally elicit lower neutralizing antibody titers; however, they induce robust cellular immune responses, particularly CD8^+^ T cell response, which is important for limiting disease severity.^98^^,^^99^ In addition to intramuscular administration, inhaled Ad-based vaccines have been developed to specifically enhance mucosal immunity to block SARS-CoV-2 entry.^100^^,^^101^ The intranasal Ad-based vaccines developed by our lab demonstrated strong immunogenicity, inducing sIgA and tissue-resident T cells in the respiratory tract, and protecting animals from viral challenges.^12^

**Bacterial and** **parasitic** **infections**: DNA vaccines targeting bacterial pathogens (*e.g.*, *Mycobacterium tuberculosis, Mtb*) and parasites (*e.g.*, *Plasmodium* spp.,^102^ *Leishmania*^103^) are under preclinical and clinical evaluation, though challenges remain in achieving robust immunity in humans. For example, several plasmid- and viral vector-based vaccines have been developed for *Mtb*. Plasmid vaccines typically encode one or multiple *Mtb* antigens, such as Ag85A, Ag85B, and Esat6.^58^^,^^104^ These vaccines generally induce suboptimal *Mtb*-specific T cell responses and only partial protective efficacies in animal models, making translation into humans difficult.

Notably, viral-vectored TB vaccines, including those based on Ad,^105^ cytomegalovirus (CMV),^64^ and modified vaccinia Ankara (MVA),^106^^,^^107^ have demonstrated potency in inducing durable and protective immunity in animal models. Among these, Ad-vectored vaccines have shown abilities in inducing potent T cell responses, particularly Th1-skewed and cytotoxic T cell responses, against *Mtb* infection in animal models.^108^^,^^109^ Several Ad-vectored candidates, including AdHu5Ag85A^110^ and ChAdOx1.85A,^111^ have entered clinical trials. The AdHu5Ag85A is a recombinant human Ad type 5 vector encoding *Mtb* antigen Ag85A.^108^ It has demonstrated safety and immunogenicity in both BCG-vaccinated and BCG-naïve individuals via intramuscular immunization.^108^ However, there is concern that pre-existing anti-Ad5 immunity may dampen the efficacy of Ad5-vectored vaccines. To address this, one solution is to use alternative Ad serotypes such as the chimpanzee Ad vector used by the ChAdOx1.85A.^111^ Efforts are still being made to further improve Ad-vector TB vaccines, including strategies such as incorporating multiple antigens to target different *Mtb* infection stages^105^ and performing airway immunization to elicit airway mucosal protection against airborne *Mtb* transmission.^108^^,^^111^

In parallel, CMV-vectored TB vaccines have also been explored.^64^^,^^112^ In one study, subcutaneous injection of rhesus CMV encoding nine *Mtb* antigens in rhesus macaques induced effector-differentiated T cell responses and reduced the overall disease burden by 68% during *Mtb* infection at one year after vaccination.^64^ In particular, one-third of vaccinated animals demonstrated sterilizing immunity in cultured tissues.^64^ Despite demonstrating remarkable efficacy in nonhuman primates, CMV-vectored TB vaccines have not entered clinical trials in humans. This is likely due to safety concerns associated with the CMV vector, including its potential for lifelong persistence in the host.

Although current viral-vectored TB vaccines have demonstrated certain protection against *Mtb* infection, none has shown superior efficacy compared to the conventional BCG vaccine. One contributing factor is pre-existing vector immunity, which can target the vector itself and reduce the expression level of the encoded antigen, thereby limiting the development of a potent T cell response against *Mtb*. Another major factor is the complex multi-stage nature of *Mtb* infection, in which different pathogenic antigens are expressed at different stages. Consequently, vaccines encoding a single antigen may fail to elicit protective immunity throughout the life cycle of *Mtb*. For this reason, viral-vectored vaccines are mainly being used as boosters following primary BCG immunization.^62^^,^^113^

### Cancer immunotherapy

DNA vaccines are being actively explored as therapeutic agents for various cancers, leveraging their ability to induce strong cytotoxic T lymphocyte (CTL) responses against tumor-associated antigens (TAAs).

**Personalized** **cancer** **vaccines (neoantigen-targeted):** Neoantigen DNA vaccines are tailored to each patient's tumor-specific mutations.^114^^,^^115^ Early-phase trials in triple-negative breast cancer (TNBC) show they are safe, feasible, and elicit strong neoantigen-specific T cell responses in ∼78% of patients, with a 36-month recurrence-free survival of ∼87.5%, compared to ∼49% historical controls.^116^ In preclinical models, “polyepitope” constructs (encoding multiple long (> 20-mer) neoantigens fused to mutant ubiquitin) triggered robust CD8 and CD4 T-cell responses.^117^ Trials are underway in glioblastoma and pancreatic carcinoma (*e.g.*, NCT03199040 in TNBC) combining neoantigen DNA vaccines with PD-L1 inhibitors.^118^^,^^119^

**Established** **targets:** TAAs (HER2, PSA, CEA, MAGE-A) DNA vaccines targeting TAAs such as HER2/neu,^120^ prostatic acid phosphatase (PAP),^121^ carcinoembryonic antigen (CEA),^122^ and MAGE-A^123^ have shown promise in clinical trials for breast, prostate, colorectal cancers, and melanoma.

pTVG-HP (encoding PAP) has been tested in Phase I/II trials, inducing durable antibody and T cell responses; long-term follow-up (15 years) showed a median overall survival of 12.3 years in castration-sensitive patients, with no vaccine-related toxicity.^123^ The pTVG-AR (targeting the ligand-binding domain of the androgen receptor) stimulated Th1 responses and extended PSA progression-free survival by ∼18 months.^124^ Combination trials (pTVG-HP + pembrolizumab) are ongoing, with results expected by December 2025.^125^

Melanoma antigen (MAGE)-A family members were identified in various human cancers, all of which have no or low expression in normal human tissues, and due to this restricted expression, the MAGE-A family represents an ideal immune therapy target.^125^ Pancreatic cancer models using multi-MAGE-A DNA vaccines eliminated chemoresistant tumors and reduced metastasis in mice.^126^ MAGE-A3 peptides have also been used as CTL-eliciting targets in clinical studies.^127^

### Veterinary medicine

DNA vaccines have found earlier and more widespread use in veterinary settings due to fewer regulatory constraints and demonstrated efficacy in multiple species. Several DNA vaccines have been approved for use in animals, including vaccines for West Nile virus^128^ in horses, IHNV in salmon,^129^ and canine melanoma.^130^ Due to their easy design and production, DNA vaccines have great potential for emerging applications. They are being developed for livestock diseases, such as foot-and-mouth disease^131^^,^^132^ and avian influenza^133^^,^^134^ with significant agricultural and economic implications.

### Pandemic preparedness and emerging pathogens

Due to their rapid design and production timeline, DNA vaccines are particularly suited for outbreak responses. The development platforms can be quickly adapted to cope with emerging threats such as novel influenza strains or coronaviruses. Once the genome of a pathogen is sequenced, expression plasmids encoding immunogenic antigens can be designed, synthesized, and validated within weeks. This allows both plasmid- and viral vector-based vaccines to be rapidly developed in response to emerging threats, such as influenza and coronaviruses. Both plasmid and viral vectored vaccines can be manufactured under good manufactured production (GMP) conditions. Plasmid-based vaccines are typically produced through bacterial fermentation and purified by established protocols. Viral vectored vaccines are usually produced in cell lines using bioreactor systems, and their production can be scaled up in bioreactors ranging from 200 to 2000 L.^135^ It is estimated that approximately 2000 doses per liter of ChAdOx1 vaccines can be produced.^136^ Thus, these scalable bioreactors can produce millions of doses if needed, highlighting the feasibility of using DNA vaccines for rapid response to global health threats.

## Limitations and challenges

Despite their numerous advantages, DNA-based vaccines face several limitations and technical hurdles that have constrained their widespread adoption, particularly in human applications. Addressing these challenges is essential to realizing the full potential of DNA vaccine platforms in both prophylactic and therapeutic settings.

### Limited immunogenicity of plasmid DNA-based vaccines in humans

One of the primary challenges for DNA vaccines is their comparatively low immunogenicity in humans. While robust immune responses have been observed in small animals and some veterinary applications, translating these results to humans has been inconsistent.^137^^,^^138^ Poor cellular and nuclear uptake of naked DNA and low antigen expression contribute to suboptimal immune activation.^139^ Human trials have often required high doses and multiple boosters to achieve meaningful responses.^140^^,^^141^ Although techniques like electroporation and gene guns improve uptake, they can be costly, uncomfortable, or technically complex, limiting scalability and field deployment.

### Concerns about genomic integration

Although rare, there is a theoretical risk of insertional mutagenesis if plasmid DNA integrates into the host genome.^142^ This risk is particularly concerning for therapeutic applications or repeated dosing.^143^^,^^144^ Enhancing vaccine efficacy could potentially lower the dose for immunization and reduce the frequency of booster administration, thereby minimizing the risk of mutagenesis.

### Limited clinical success and regulatory approval of plasmid DNA-based vaccines

Although viral vector DNA-based vaccines are approved for various applications, only a few plasmid DNA vaccines have received approval for use in humans, with most successes confined to veterinary medicine.^144^ One way to improve their utility is to use them as boosting vaccines. Because viral vector DNA-based vaccines require heterologous boosting, plasmid DNA-based vaccines may be good for boosting.

### Potential for anti-DNA immune responses

There is a theoretical concern that repeated administration of DNA vaccines could induce anti-DNA antibodies or inflammatory responses, particularly in individuals predisposed to autoimmune diseases. However, clinical data have not shown this to be a major issue to date.^145^

## Recent innovations and future directions

Ongoing research and technological advancements have significantly expanded the potential of DNA-based vaccines. While early limitations such as low immunogenicity and inefficient delivery once hindered progress, recent innovations in vector design, delivery technologies, and immunomodulation strategies are revitalizing interest in this platform. The field is also evolving toward broader clinical applications and global readiness for emerging threats.

### Optimized antigen expression

To enhance antigen expression for immune activation, several strategies have been employed in the design of antigen expression cassettes. First, strong mammalian promoters (*e.g.*, CMV, chicken-beta-actin) and enhancer elements have been used to increase transcription levels.^146^^,^^147^ Second, codon optimization which aligns antigen gene sequences with host cell tRNA abundance has been used to improve translation efficiency.^148^^,^^149^ Third, certain amino acids, such as proline, can be introduced to increase protein stability.^150^ Finally, avoiding bacterial DNA sequences in viral vectors or using minicircle DNA has been used to enhance antigen expression.^151^

### Next-generation delivery systems

Innovations in DNA delivery systems may help overcome barriers to cellular uptake and antigen expression. Although not clinically approved, advanced electroporation devices are used to enhance DNA vaccine efficacy.^152^

Microneedle arrays allow minimally invasive, self-administered, and targeted dermal delivery with high APC density. Microneedles offer a minimally invasive platform to deliver DNA vaccines into the epidermis and dermis, where immune cells are abundant. It is a painless and user-friendly delivery system, suitable for mass immunization campaigns. It is currently under investigation for delivering both prophylactic^153^ and therapeutic DNA vaccines.^154^

Lipid nanoparticles (LNPs) have been widely used in mRNA vaccines, especially during the COVID-19 pandemic.^5^ Biodegradable nanoparticles (*e.g.*, liposomes,^31^ polymeric particles,^155^ lipid–polymer hybrid^79^) have emerged as promising vehicles for DNA vaccine delivery as well. These LNP carriers protect DNA from enzymatic degradation and facilitate cellular uptake through endocytosis. They can be engineered for targeted delivery to immune cells through surface modification.^156^^,^^157^

### Heterologous prime-boost strategies

As aforementioned, pre-existing vector-specific immunity could reduce the efficacy of homologous booster doses. Therefore, a heterologous prime-boost regimen is a feasible approach to achieve potent vaccine efficacy. For example, viral vector DNA-based vaccines, such as those delivered with the first-generation Ad vectors, can elicit excellent T cell and humoral responses when used for priming. A second dose encoding the same antigen, regardless of whether it is delivered in the form of another viral vector, plasmid DNA, mRNA, or protein, can be tested for boosting immunogenicity. In fact, this approach is being explored in SARS-CoV-2,^158^ HIV,^159^ HPV,^160^ Ebola^55^ and tuberculosis^62^ vaccines.

### Development of single-dose vaccines

Single-dose vaccine does not require boosting. It has many advantages over other vaccine regimens, such as simplified logistics since one clinic visit is needed, improved compliance, and cost-effectiveness. Most vaccines cannot be used as single-dose vaccine because the delivered antigens may not last long enough, or the vaccines are not potent enough to induce strong long-lasting immune responses. HDAd vectors have the great potential for being used to develop single-dose vaccines. These vectors have high efficiency in the delivery of genes^161^, ^162^, ^163^, ^164^, ^165^ or shRNAs.^166^ Transgene expression from this type of vector lasts longer compared to the conventional Ad vectors.^167^ Recent studies in animal models have demonstrated that a single-dose HDAd-based COVID-19 vaccine can induce protective immunity against SARS-CoV-2 challenge.^11^^,^^12^ Vaccinated mice showed no detectable SARS-CoV-2 in the upper and lower airways, similar to the observations in mice vaccinated with a prime boost dose of mRNA-1273^168^ or BNT162b2.^169^

### Expansion into therapeutic vaccination

Because of their simplicity in engineering, DNA vaccines are increasingly being developed for therapeutic applications. Cancer immunotherapy is a current focus, with clinical trials underway for melanoma,^170^ prostate cancer,^121^ cervical cancer,^171^ and glioblastoma.^172^ Chronic infections like hepatitis B,^173^ HIV,^174^ and HPV-associated^171^ diseases are key targets for therapeutic DNA vaccination aiming to restore or enhance cellular immunity.

### Artificial intelligence and synthetic biology

Emerging tools in AI-driven antigen prediction and synthetic biology are revolutionizing antigen discovery and vaccine design. Machine learning algorithms can identify novel epitopes with high immunogenic potential.^175^ Synthetic platforms allow for rapid re-engineering of vaccine constructs to match circulating pathogen variants.^175^

### Pandemic and global health preparedness

The COVID-19 pandemic accelerated interest in DNA vaccines due to their rapid development timelines, thermal stability, and ease of manufacture. Both viral vector^7^^,^^8^ and plasmid (*e.g.*, ZyCoV-D^9^) DNA-based COVID-19 vaccines received emergency or full regulatory approval. These platforms are now central to “plug-and-play” pandemic response models, with pre-established backbones ready for antigen swap-in to cope with new emerging pathogens.

## Conclusion

DNA-based vaccines have emerged as a powerful and versatile platform with the potential to transform both preventive and therapeutic immunization strategies. Their ability to induce robust humoral and cellular immune responses, combined with technological and manufactural advantages, makes them particularly well-suited for responding to emerging infectious threats and for application in precision medicine.

Over the past decades, advances in vector engineering, delivery technologies, and adjuvant systems have significantly enhanced the immunogenicity and clinical promise of DNA vaccines. Their successful deployment in veterinary medicine and growing momentum in human clinical trials—most notably during the COVID-19 pandemic—underscore their readiness for broader application. Moreover, the expanding use of DNA vaccines in cancer immunotherapy and chronic infectious diseases demonstrates the platform's therapeutic versatility.

Despite these advances, important challenges remain. Issues such as relatively low immunogenicity in humans, inefficient delivery without specialized devices, and limited regulatory approvals must be addressed to fully unlock the platform's potential. Continued innovation in delivery methods, molecular adjuvants, and personalized vaccine design—alongside increasing global investment and regulatory engagement—will be crucial for the next generation of DNA-based vaccines.

In conclusion, DNA vaccines represent a promising and adaptable technology at the frontier of vaccinology. As scientific and technological barriers continue to be overcome, DNA-based immunization strategies are poised to become an integral part of the global vaccine arsenal, with profound implications for infectious disease control, cancer therapy, and public health preparedness. Continued innovation in vector engineering, delivery technologies, and immunomodulation will likely improve clinical efficacy and enable broader application across infectious diseases, oncology, and autoimmunity. Moreover, their ease of customization and favorable logistics position DNA vaccines as a key component of global health security and personalized medicine in the years ahead.

## Funding

This work is partially supported by 10.13039/501100000024CIHR grants to J.H. and J.L. and a 10.13039/501100000023Canadian Government grant, NFRFE-2021-00713 to J.H.

## CRediT authorship contribution statement

**Zhichang Peter Zhou:** Writing – review & editing, Writing – original draft. **Ziyan Rachel Chen:** Writing – review & editing, Visualization. **Ranmal Avinash Bandara:** Writing – review & editing. **Rongqi Duan:** Writing – review & editing. **Huibi Cao:** Writing – review & editing. **Jun Liu:** Writing – review & editing, Funding acquisition. **Jim Hu:** Writing – review & editing, Writing – original draft, Funding acquisition, Conceptualization.

## Conflict of interests

Jim Hu is a member of the *Genes & Diseases* Editorial Board. To minimize bias, he was excluded from all editorial decision-making related to the acceptance of this article for publication. The remaining authors declare no conflict of interests.
